# Development and validation of a practical clinical risk prediction model for post‐endoscopic retrograde cholangiopancreatography pancreatitis

**DOI:** 10.1002/deo2.355

**Published:** 2024-03-25

**Authors:** Zhao Wu Meng, Yibing Ruan, Stacey Fisher, Kirles Bishay, Millie Chau, Megan Howarth, Shane Cartwright, Yen‐I Chen, Elijah Dixon, Steven J. Heitman, Darren R. Brenner, Nauzer Forbes

**Affiliations:** ^1^ Department of Community Health Sciences University of Calgary Calgary Canada; ^2^ Department of Medicine Division of Gastroenterology and Hepatology University of Calgary Calgary Canada; ^3^ Department of Oncology Cumming School of Medicine University of Calgary Calgary Canada; ^4^ Department of Cancer Epidemiology and Prevention Research Cancer Care Alberta, Alberta Health Services Calgary Canada; ^5^ Ottawa Hospital Research Institute Ottawa Canada; ^6^ Department of Medicine, Division of Gastroenterology and Hepatology McGill University Health Centre Montreal Canada; ^7^ Department of Surgery University of Calgary Calgary Canada

**Keywords:** adverse event, endoscopy, ERCP, pancreatitis, prediction

## Abstract

**Background:**

Pancreatitis following endoscopic retrograde cholangiopancreatography (ERCP) can lead to significant morbidity and mortality. We aimed to develop an accurate post‐ERCP pancreatitis risk prediction model using easily obtainable variables.

**Methods:**

Using prospective multi‐center ERCP data, we performed logistic regression using stepwise selection on several patient‐, procedure‐, and endoscopist‐related factors that were determined a priori. The final model was based on a combination of the Bayesian information criterion and Akaike's information criterion performance, balancing the inclusion of clinically relevant variables and model parsimony. All available data were used for model development, with subsequent internal validation performed on bootstrapped data using 10‐fold cross‐validation.

**Results:**

Data from 3021 ERCPs were used to inform models. There were 151 cases of post‐ERCP pancreatitis (5.0% incidence). Variables included in the final model included female sex, pancreatic duct cannulation, native papilla status, pre‐cut sphincterotomy, increasing cannulation time, presence of biliary stricture, patient age, and placement of a pancreatic duct stent. The final model was discriminating, with a receiver operating characteristic curve statistic of 0.79, and well‐calibrated, with a predicted risk‐to‐observed risk ratio of 1.003.

**Conclusions:**

We successfully developed and internally validated a promising post‐ERCP pancreatitis clinical prediction model using easily obtainable variables that are known at baseline or observed during the ERCP procedure. The model achieved an area under the curve of 0.79. External validation is planned as additional data becomes available.

## INTRODUCTION

Endoscopic retrograde cholangiopancreatography (ERCP) is a mainstay of pancreaticobiliary endoscopy^1^ but has the highest adverse event rates of all commonly performed endoscopic procedures.^2^, ^3^ Of these, post‐ERCP pancreatitis (PEP) is the most common serious adverse event and can lead to significant morbidity or mortality.^4^, ^5^, ^6^ PEP occurs in 5%–10% of ERCP patients^7^ and 15% or more of high‐risk patients.^8^ Over $200 million annually is spent on direct consequences of PEP in the United States.^9^, ^10^, ^11^

Several strategies mitigate the risk of PEP, including rectal non‐steroidal anti‐inflammatory drugs (NSAIDs), aggressive intravenous fluids, and prophylactic pancreatic duct stents.^8^, ^12^ However, each strategy is associated with perceived and/or actual disadvantages, leading to suboptimal usage.^13^ Given this, and given that the underlying risk of PEP often cannot be mitigated due to unmodifiable patient‐related factors, a reliable risk prediction tool could assist with complex medical decision‐making regarding prophylaxis and/or monitoring. From a patient perspective, such a tool could help patients understand their individualized PEP risk and assist them in their informed decision‐making process.

Previous attempts at devising robust PEP risk calculators have been limited by various factors including retrospective data collection,^14^ self‐reporting, lack of validation,^15^ and suboptimal performance.^16^ Thus, we aimed to develop and validate an accurate PEP prediction tool for eventual application on an individual patient level using robust, bias‐limited, and multi‐center prospective data.

## METHODS

### Study design, setting, and patient population

We conducted our study according to the Transparent Reporting of a Multivariable Prediction Model for Individual Prognosis or Diagnosis guidelines.^17^ Data used to inform risk prediction models were obtained via a prospective, multi‐center ERCP data collection initiative (NCT04259580 and REB18‐0410).^18^ Patients were eligible for inclusion if they were adults undergoing ERCP for any biliary indication. Trained observers recorded intra‐procedural data at each sub‐center in real‐time (with no self‐reporting by endoscopists). Data centers were chosen to reflect a mix of tertiary and community centers and to represent endoscopists of various experience levels. All data were stored securely.^19^

### Details of ERCPs

All procedures were performed by endoscopists having performed at least 1000 ERCPs or by trainees under their supervision. Wire‐guided cannulation was initially attempted in all cases as per standard of care (as opposed to contrast‐assisted cannulation). The performance and timing of pre‐cut sphincterotomy were left to the discretion of the endoscopist performing or supervising the procedure, as was the placement of biliary stents and/or prophylactic pancreatic stents, in order to have the results represent routine ERCP practice. Any patients in whom biliary stents were placed underwent sphincterotomy prior to placement.

### Model variables

Variables considered for inclusion included established PEP risk factors such as age, sex, history of acute pancreatitis or PEP, non‐dilated bile duct, normal bilirubin, papilla morphology, difficult cannulation (attempts for greater than 5 min or greater than five attempts), pancreatic sphincterotomy, and trainee involvement, among others,^2^, ^3^, ^20^ and potentially novel risk factors such as cumulative trainee hands‐on time, number and extent of pancreatic duct cannulations, and presence of a biliary stricture, among others.

### Outcome definition and attribution

Protocolized 30‐day follow‐up was obtained via direct patient interview and review of available medical records, thus allowing for prospective outcome ascertainment to inform the models. PEP was defined as characteristic abdominal pain occurring following ERCP that resulted in either admission of, or prolongation of admission by, a minimum of two nights, and that was associated with serum pancreatic enzyme values >3 times the upper limit of normal and/or imaging findings indicative of acute pancreatitis.^21^ A schema was used to designate probabilities of causal attribution between events meeting the above definition and index ERCPs,^22^ with two independent raters providing attributions and disagreements resolved by consensus. Pancreatitis events deemed to be either *definitely* or *probably* related to an index ERCP were considered to represent PEP, with events deemed *possibly related*, *unlikely to be related*, *unrelated*, or *unclassifiable* all excluded.^22^ Severity of PEP was determined by the consensus and ASGE lexicon definitions,^21^, ^23^ with mild PEP resulting in hospitalization of up to 3 days, moderate PEP resulting in hospitalization of between 4 and 10 days, and severe PEP meeting any of the following criteria: hospitalization of more than 10 days, development of pseudocyst or necrosis, or surgical or percutaneous intervention.

### Statistical analysis

Given an anticipated 3000 procedures, we expected 150 cases of PEP assuming a baseline incidence of 5%.^18^ We planned to use all cases for model development with subsequent internal validation via bootstrapping.^24^ According to the “rule of 10” cases per degree of freedom,^24^ we estimated that the data would support an algorithm with a maximum of 15 degrees of freedom.

We started with univariate binary logistic regression on each variable. The starting model consisted of sex (a well‐known PEP risk factor) and pancreatic duct cannulation (largest z score on univariate analysis without significant missing data). We then proceeded with a stepwise selection approach by deleting/ re‐adding variables using an alpha of 0.2. No interaction terms were included given their low yield and degree of freedom constraints. Variables were assessed for clinical importance, statistical predictive ability, parsimony, and feasibility of collection in practice before inclusion in the final predictive model.

All available data were used for model development, with internal validation based on bootstrapped data.^24^ Calibration was assessed by plotting the observed proportions of events against the predicted probabilities, stratified by decile of predicted probability. Discrimination was performed by assessing the area under the receiver operating characteristic curve statistic. Continuous variables were assessed as both continuous and dichotomized using clinically relevant cut‐offs (e.g., age < 45 years vs. ≥ 45 years) during internal validation. If performance characteristics were similar, the use of dichotomized variables in the final model was preferred to optimize usability. Our final model was the model that exhibited the best performance as per the Bayesian information criterion (BIC) and Akaike's information criterion (AIC).^25^ Statistical analyses were carried out using Stata 16.0 (StataCorp).

## RESULTS

From September 1, 2019, to January 16, 2022, patient‐, procedure‐, and endoscopist‐level data from 3021 consecutive/ unselected biliary ERCP procedures in the prospective registry were captured from four centers in Canada (four endoscopy units, 21 endoscopists). After duplicate reviewers applied outcome definitions and causal attribution criteria determined a priori,^22^ there were 151 (5.0%) cases of definite or probable PEP. Of these, five cases (3.3%) met the definition of moderate or severe PEP, with three of these requiring admissions of between 4 and 10 days without other local or systemic complications (moderate PEP), one developing a pancreatic pseudocyst requiring endoscopic drainage (severe PEP), and one requiring intensive care admission due to pancreatic necrosis and multi‐organ failure (severe PEP). Baseline characteristics describing study patients (with and without PEP) and their ERCP procedures are provided in Table 1.

**TABLE 1:** Baseline characteristics of patients and procedures.

	No PEP (*n* = 2870)	PEP (*n* = 151)	*p*‐value
Sex % female (*n*)	50.2 (1438)	57.0 (86)	0.10
Mean age (SD)	61.21 (17.55)	53.13 (17.83)	< 0.001
Mean Charlson Comorbidity Index (SD)	3.45 (2.88)	2.25 (2.60)	< 0.001
Patient disposition			
Inpatient % (*n*)	52.6 (1508)	50.0 (75)	0.68
Outpatient % (*n*)	47.4 (1361)	50.0 (75)	
Trainee involved in ERCP % (*n*)	66.2 (1900)	70.0 (105)	0.33
Procedural indication			
Suspected or known CBD stones % (*n*)	40.6 (1165)	37.7 (57)	0.49
Suspected neoplasm resulting in biliary obstruction % (*n*)	19.7 (565)	25.2 (38)	
Other (including stent exchanges, repeat procedures, etc.) % (*n*)	39.7 (1140)	37.1 (56)	
Native papilla status % (*n*)	63.9 (1766)	81.0 (111)	< 0.001
Cannulation attempts			
>10% (*n*)	9.4 (236)	31.7 (38)	< 0.001
6%–10% (*n*)	9.4 (237)	18.3 (22)	
3%–5% (*n*)	23.0 (580)	25.8 (31)	
1%–2% (*n*)	58.2 (1464)	24.2 (29)	
Mean cannulation time, minutes (SD)	4.94 (7.33)	11.57 (11.16)	< 0.001
Pre‐cut sphincterotomy % (*n*)	12.3 (340)	36.0 (49)	< 0.001
PD cannulated % (*n*)	19.9 (543)	53.0 (71)	< 0.001
Double wire technique used % (*n*)	11.0 (289)	27.9 (34)	< 0.001
Pancreatogram performed % (*n*)	7.4 (211)	25.3 (38)	< 0.001
Formal biliary sphincterotomy (with deep guidewire access) % (*n*)	59.4 (1555)	74.4 (90)	< 0.001
Balloon sphincteroplasty % (*n*)	16.6 (459)	13.6 (18)	0.35
Mean CBD size, mm (SD)	9.70 (3.14)	8.91 (2.72)	0.002
Biliary stricture present during ERCP % (*n*)	25.3 (644)	43.1 (50)	< 0.001
Failure of cannulation of targeted duct % (*n*)	4.3 (123)	6.0 (9)	0.33
CBD stent placed			
None % (*n*)	72.3 (1950)	54.9 (73)	< 0.001
Metal % (*n*)	10.2 (282)	18.8 (25)	
Plastic % (*n*)	18.6 (512)	25.6 (34)	
PD stent placed % (*n*)	7.1 (205)	11.9 (18)	0.04
Overall procedure time, minutes (SD)	23.28 (15.59)	31.74 (17.86)	< 0.001
Trainee hands‐on time, minutes (SD)	16.38 (11.09)	19.39 (13.2)	0.02
Trainee completed procedure independently % (*n*)	60.8 (1076)	38.0 (38)	0.02
Rectal NSAID given for PEP prophylaxis % (*n*)	41.4 (1153)	61.9 (91)	< 0.001

Abbreviations: CBD, common bile duct; ERCP, endoscopic retrograde cholangiopancreatography; PD, pancreatic stent; PEP, post‐ERCP pancreatitis; SD, standard deviation.

### Univariate analysis

**FIGURE 1:**
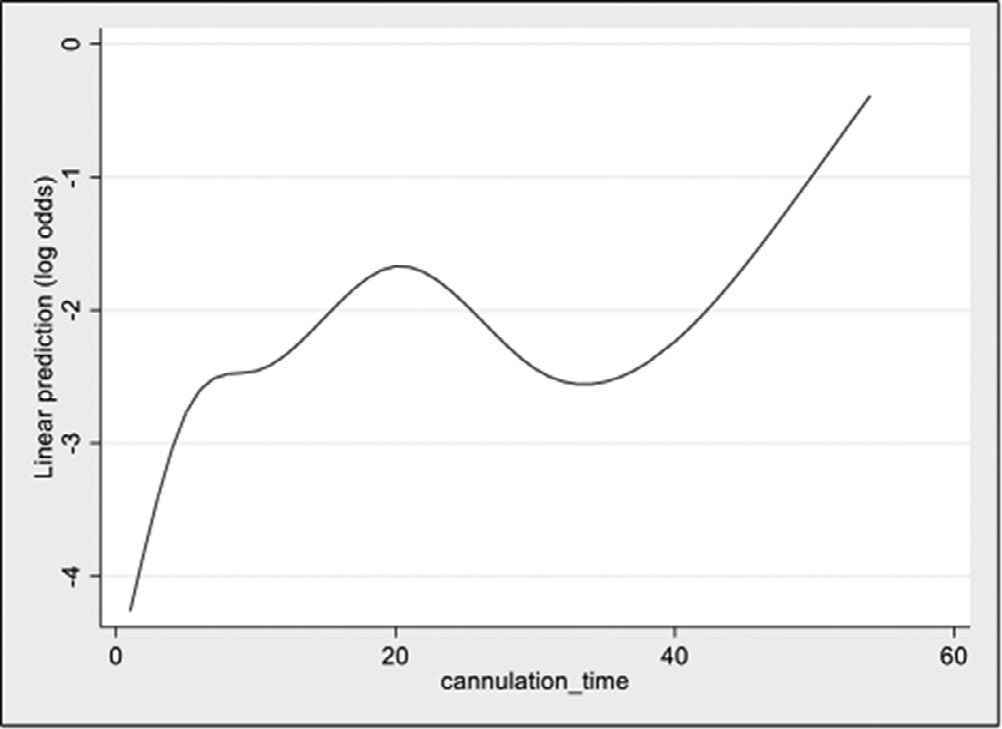
Restricted cubic spline of cannulation time (in minutes).

Univariate analysis was performed to explore how potential predictive factors were associated with PEP (Table 2). Suspected sphincter of Oddi dysfunction as a procedural indication, native papilla status, normal (Type I) papilla morphology, flat (Type II) papilla morphology, pancreatic stent (PD) cannulation, pancreatogram, precut sphincterotomy, double wire technique, multiple common bile duct cannulation attempts, and prolonged cannulation time (>5 and >10 min), PD stenting, indomethacin use, and new pain after procedure were all independently associated with PEP risk. History of sphincter of Oddi dysfunction was excluded from further analysis due to low case numbers leading to low levels of confidence in the point estimate of associated PEP risk. Age was retained as a discreet variable after restricted cubic spline analysis did not demonstrate significant transition points. Cannulation time was initially captured as a continuous variable but transformed into a categorical variable (<10, 10–19, 20–29, and ≥ 30 min) after visual analysis of a restricted cubic spline plot demonstrated multiple transition points (Figure 1).

**TABLE 2:** Results of univariate binary regression.

Variable	OR	95% CI	*p*‐value
Female (vs. male)	1.31	0.94–1.83	0.11
Age (integer)	0.98	0.97–0.98	<0.001
History of acute pancreatitis (vs. none)	0.84	0.30–2.32	0.74
Suspected SOD (vs. none)	9.73	2.89–32.70	<0.001
Procedural indication stones (vs. all others)	0.90	0.65–1.26	0.55
Presence of CBD stricture (vs. none)	0.74	0.34–1.60	0.45
Native papilla (vs. previous sphincterotomy)	2.42	1.56–3.73	<0.001
Normal Type I papilla morphology (vs. all others)	1.41	1.02–1.93	0.03
Flat Type II papilla morphology (vs. all others)	1.99	1.09–3.60	0.02
PD cannulation (vs. none)	4.14	2.98–5.74	<0.001
Pancreatogram (vs. none)	3.76	2.67–5.31	<0.001
Minor papilla cannulation (vs. none)	6.32	3.76–10.65	<0.001
Pre‐cut sphincterotomy (vs. none)	3.64	2.61–5.08	<0.001
Double wire technique (vs. none)	2.89	1.98–4.23	<0.001
≥3 cannulation attempts (vs. fewer)	4.10	2.72–6.18	<0.001
>5 min cannulation time (vs. shorter)	4.11	2.91–5.83	<0.001
>10 min cannulation (vs. shorter)	3.61	2.63–4.96	<0.001
Cannulation time			
10–19 min (vs. <10 min)	4.61	2.86–7.42	<0.001
20–29 min (vs. <10 min)	5.39	2.64–11.00	<0.001
≥30 min (vs. <10 min)	3.47	2.38–5.08	<0.001
Biliary sphincterotomy	1.98	1.31–3.01	0.001
Sphincteroplasty (vs. none)	0.80	0.49–1.30	0.37
Cholangioscopy performed (vs. not)	1.04	0.39–2.74	0.69
Prophylactic PD stent placed (vs. none)	1.53	0.79–2.98	0.21
Rectal NSAID given (vs. not)	2.20	1.59–3.05	<0.001
New abdominal pain after procedure^33^ (vs. none)	3.91	2.60–5.87	<0.001

Abbreviations: CBD, common bile duct; CI, confidence interval; OR, odds ratio; NSAID, non‐steroidal anti‐inflammatory drug; PD, pancreatic stent; SOD, sphincter of Oddi dysfunction.

### Multivariable analysis

**FIGURE 2:**
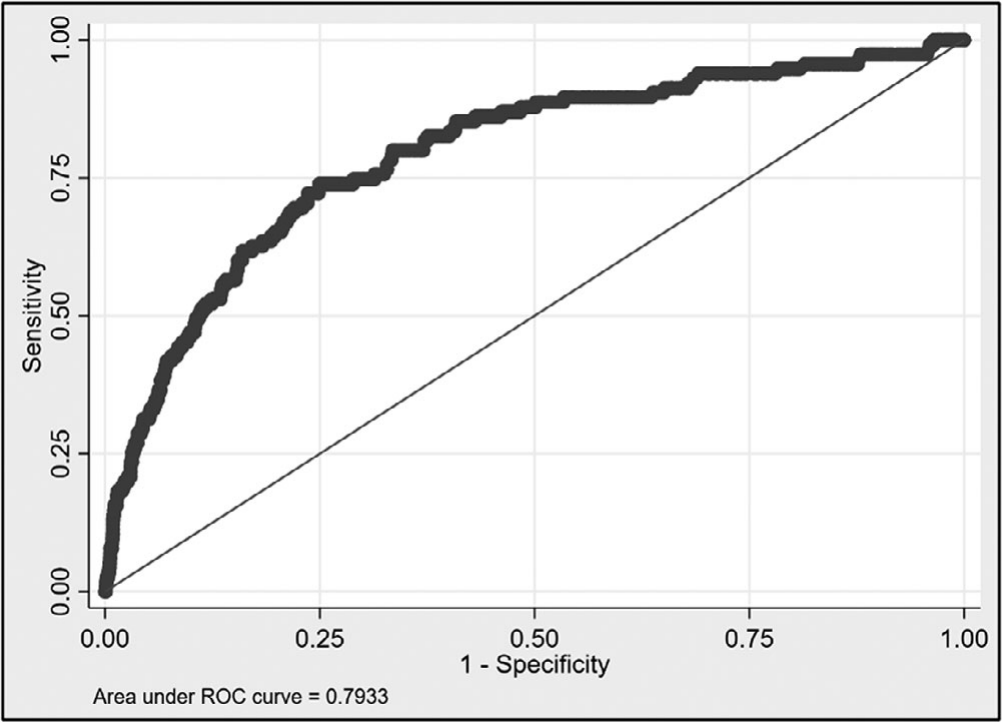
Receiver operating characteristic curve of the final predictive model.

Variables included in the final prediction model were sex, age, native papilla status, pancreatic duct cannulation (of any depth or frequency), pre‐cut sphincterotomy (including trans‐pancreatic or suprapapillary pre‐cuts but considering needle‐knife papillotomy as a pre‐cut), cannulation time, the presence of a biliary stricture, and pancreatic duct stenting, with 10 degrees of freedom (Table 3). Increasing age was protective, as was the placement of a pancreatic duct stent, while all other included variables were associated with higher PEP risk. This final model had an AIC and BIC of 822.28 and 886.77, respectively. The model had an area under the curve (AUC) of 0.79 (Figure 2). The model performed incrementally better with the inclusion of a flat (Type II) papilla morphology,^20^ with corresponding AIC, BIC, and AUC values of 819.30, 889.67, and 0.80, respectively. However, papilla morphology was ultimately not included in the final model given the established moderate inter‐observer variability associated with its description in ERCP. ^26^ The model was also reassessed after performing multiple imputations of missing data, with model performance very similar to the pre‐multiple imputation model, indicated by AIC, BIC, and AUC values of 1067.28, 1133.39, and 0.77, respectively.

**TABLE 3:** Odds ratios associated with the final clinical prediction model developed using multivariable logistic regression (10 degrees of freedom).

Variables	OR	95% CI	β	*p*‐value
Female sex (vs. male)	1.45	0.97–2.18	0.38	0.07
Age (per increasing year)	0.98	0.97–0.99	‐0.02	<0.001
Native papilla (vs. previous sphincterotomy)	1.95	1.10–3.46	0.67	0.02
PD cannulation (vs. not)	3.56	2.27–5.59	1.27	<0.001
Pre‐cut sphincterotomy	1.41	0.84–2.38	0.35	0.19
Cannulation time				
10–19 min (vs. others)	2.23	1.28–3.89	0.80	0.01
20–29 min (vs. others)	2.10	0.89–4.92	0.74	0.09
≥ 30 min (vs. shorter)	2.42	1.32–4.44	0.88	0.00
CBD stricture present (vs. none)	2.33	1.52–3.56	0.84	<0.001
Prophylactic pancreatic duct stent placed (vs. none)	0.53	0.29–0.98	‐0.64	0.04

Abbreviations: CBD, common bile duct; CI; confidence interval; OR, odds ratio; PD, pancreatic stent.

Y‐intercept (baseline odds) = 0.029.

### Internal validation

**FIGURE 3:**
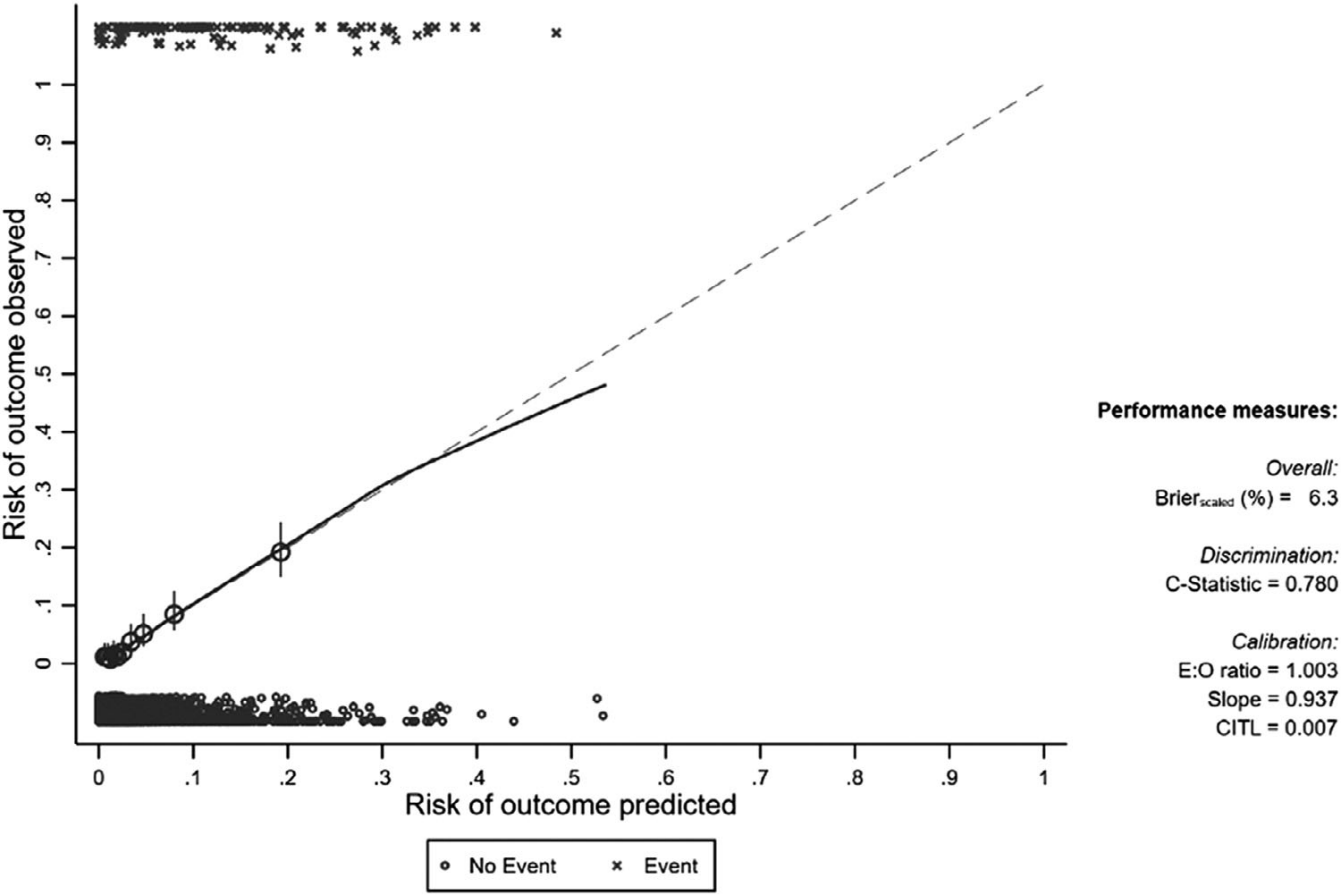
Internal validation of the predictive model using 800 bootstrapped replicates.

The entire dataset was bootstrapped using 800 bootstraps, with results provided in Figure 3. Calibration, as measured by an expected to observed ratio, was 1.003, with a corresponding slope of 0.937. Discrimination as measured by the C‐statistic was 0.78.

### Application of the model using clinical examples

Using the final multivariable model, probabilities of PEP, reflected as percentages, were calculated by setting arbitrary values for all variables for several illustrative clinical scenarios, as follows. For an 18‐year‐old male with no other risk factors, the predicted risk of PEP would be 1.9%. For an 80‐year‐old male with no other risk factors, the predicted risk of PEP would be 0.4%. For an 18‐year‐old female with a native papilla, the predicted risk would be 5.2%. If the same female were to experience inadvertent pancreatic duct cannulation, the risk would increase to 16.4%. By receiving a prophylactic pancreatic duct stent, the same female patient's predicted risk of PEP would then be reduced to 9.4%. The highest‐risk scenario calculated by the model (an 18‐year‐old female with a native papilla and a bile duct stricture who requires a pre‐cut sphincterotomy and undergoes inadvertent pancreatic duct cannulation during a > 30‐min cannulation without subsequent placement of a PD stent) would be associated with a predicted PEP risk of 60.9%.

## DISCUSSION

In this study, a simple but accurate clinical prediction model for post‐ERCP pancreatitis was developed and validated using bias‐limited multi‐center prospective data. The model was designed to be applied in real time during the performance of ERCP, and in so doing, could provide early PEP risk assessment that could potentially alter monitoring and/or management strategies. By incorporating eight easily obtainable clinical variables to inform the model, it achieved an AUC of 0.79, which is on the cusp of acceptable and excellent accuracy.

Various mechanisms for PEP have been proposed, including mechanical obstruction of the papilla and pancreatic sphincter by instrumentation, hydrostatic injury from injection of contrast or water, chemical or allergic injury from contrast, and edema due to thermal injury secondary to electrocautery.^27^ Despite our understanding of these mechanisms, the ability to predict clinically significant PEP at the individual patient level remains poor. Eight clinical variables were ultimately included in our model, which performed well in its validation. A notable omission was the administration of rectal NSAIDs. While our univariate analysis revealed a strong correlation between indomethacin use and PEP, it did not perform well in the multivariable analysis, likely due to an element of selection bias governing its clinical use in our cohort. Outside of a randomized trial, there is a possibility of selection bias (and the subsequent possible interpretation of results as reverse causality). For example, if NSAIDs were administered more frequently to patients who were deemed to be at high risk of PEP (arbitrarily, 15%), and they effectively reduced this risk by one‐third, to 10%, this would still be double the baseline risk of PEP and could create the false impression that NSAIDs increase PEP risk. For this reason, we judged that the inclusion of NSAID administration in our model could potentially be misleading, and we confirmed our suspicion of selection bias in supplementary analyses (Supporting Information). However, given the preponderance of evidence supporting the use of rectal NSAIDs, both for all‐comers and high‐risk patients,^8^ we strongly support its widespread use in the absence of contraindications.

The presence of several variables related to pancreatic duct manipulation should pathophysiologically predict PEP, including pancreatic duct cannulation, the number of cannulations, the depth of cannulations, side branch cannulation, the use of the double wire technique, and the performance of a pancreatogram. Despite each of these variables exhibiting strong signals in univariate analysis, many did not perform well in the multivariable analysis, likely due to high levels of collinearity. As such, the final model included only pancreatic duct cannulation as an input variable (any cannulation, regardless of frequency, depth, or location in the PD). Prophylactic pancreatic duct stenting is known to be protective against PEP,^8^ and this was also demonstrated in our study. Given our understanding of the mechanisms of PEP above, our model therefore makes intuitive sense.

Previous models have been developed for risk prediction around ERCP‐related outcomes.^14^, ^15^, ^16^, ^28^, ^29^ Some have been limited by a low number of input cases, whereas others have lacked granularity in input variables, and still others have relied upon self‐reporting and/or retrospective data collection, which can introduce important sources of bias into models. Crucially, many prior models have been limited to data from single centers, an approach that limits generalizability and applicability. Thus, by using bias‐limited multi‐center prospective data acquired at both tertiary care and community centers, we aimed to mitigate some of these issues. The use of prospective data is also preferable as it eliminates recall bias. By collecting data in real‐time during the procedure, we minimize endoscopists’ influence on data collection and mitigate recall bias. Furthermore, using all available data for model synthesis and bootstrapping for subsequent validation increases performance. The results of internal validation also suggest that there was minimal overfitting, as measured by both discrimination and calibration.

There are also some important limitations associated with our model. First, while data from both academic and community centers were included, the majority of ERCPs informing the model were completed at high‐volume tertiary centers, and the overall number of procedures is still low relative to the widespread performance of ERCPs. This could theoretically limit the model's generalizability overall, and therefore, external validation should be performed in various settings. Secondly, logistic regression has traditionally been the most used method for prediction modeling; however, machine learning (ML) has increasingly been considered an alternative, and its lack of application could be considered a limitation. While ML holds potential, there are also associated pitfalls, such as overfitting of data and lack of ability to robustly assess the reliability of predictors (i.e., via calibration).^30^, ^31^ A recent systematic review demonstrated no benefit to ML over logistical regression when creating prediction models.^32^ Given this, we are reassured by our approach. Thirdly, the choice of bootstrapping, as opposed to data splitting, for internal validation, could be questioned. We opted for the former approach given increasing support for bootstrapping in recent prediction modelling literature.^24^ Fourthly, our prediction tool, while able to accurately predict PEP risk on a patient level, does not make any subsequent recommendations based on this prediction. Robust practice recommendations (on prophylactic measures, monitoring, and/or management) based on patients’ predicted risks could impact practice even more meaningfully but would require dedicated cost‐effectiveness analyses to evaluate the thresholds above/below which actions would be justified. This should be the focus of future prediction tools. Additionally, it would arguably be of even greater value to design and validate a tool that can predict cases of moderate or severe PEP. While we attempted to assess these data, we observed only five cases of moderate‐severe PEP, and therefore, the event rate was too low to study a prediction tool in this population. This should also be a focus for future investigation. Finally, we were unable to assess the impacts of post‐procedural aggressive intravenous fluids, an intervention shown to decrease the incidence of PEP. No patients in our cohort received 24‐h prophylactic fluid regimens due to the rare adoption of this intervention in Western practices. However, now that guidance suggests implementing this intervention where possible, it will be important to clarify its protective effect in future risk prediction models.

Overall, we successfully developed and validated a PEP clinical prediction model using variables that are easily assessed during the routine performance of ERCP. External validation is required prior to widespread clinical use, ideally in a prospective setting as well. Furthermore, future studies should pair high‐fidelity prediction models with appropriately performed cost‐effectiveness analyses in order to allow for recommendations to guide practice during and after ERCP based on individualized patient risks.

## CONFLICT OF INTEREST STATEMENT

Nauzer Forbes has been a consultant for Boston Scientific, has been on the speaker's bureau for Pentax Medical and Boston Scientific, and has received research funding from Pentax Medical. The rest of the authors declare no conflict of interest.

## Supporting information


**TABLE S1**: Background factors among patients receiving peri‐ (pre‐, peri‐, or post‐) procedural non‐steroidal anti‐inflammatory drugs (NSAIDs) versus not receiving peri‐procedural NSAIDs.
