# Supplementary material for: Development and validation of a practical clinical risk prediction model for post‐endoscopic retrograde cholangiopancreatography pancreatitis
Source: DEN Open. 2024 Mar 25;4(1):e355. doi: 10.1002/deo2.355 (PMC10962486; doi:10.1002/deo2.355)
Supplement: Supplementary file 1 — TABLE S1: Background factors among patients receiving peri‐ (pre‐, peri‐, or post‐) procedural non‐steroidal anti‐inflammatory drugs (NSAIDs) versus not receiving peri‐procedural NSAIDs. [file DEO2-4-e355-s001.docx]

**SUPPORTING INFORMATION**

**S1. Background factors among patients receiving peri- (pre-, peri-, or post-) procedural NSAIDs vs. not receiving peri-procedural NSAIDs.**

| Variable | Received Indomethacin (1244/3021) | Did not receive rectal indomethacin (1688/3021) | P value |
| --- | --- | --- | --- |
| PEP | 91/147 | 56/147 | <0.001 |
| Female | 774/1478 | 704/1478 | <0.001 |
| Native papilla | 965/1828 | 863/1828 | <0.001 |
| Pancreaticogram | 157/240 | 83/240 | <0.001 |
| Precut | 218/375 | 157/375 | <0.001 |
| PD stent | 155/215 | 60/215 | <0.001 |
| Prophylactic PD stent | 100/133 | 33/133 | <0.001 |
| 10 minute cannulation | 415/806 | 391/806 | <0.001 |
| Stricture present | 220/675 | 455/675 | <0.001 |
| PD cannulated | 362/599 | 237/599 | <0.001 |

*89/3021 patients missing data on indomethacin use
